# Mesenchymal Stem Cells and Platelet Gel Improve Bone Deposition within CAD-CAM Custom-Made Ceramic HA Scaffolds for Condyle Substitution

**DOI:** 10.1155/2013/549762

**Published:** 2013-09-01

**Authors:** L. Ciocca, D. Donati, S. Ragazzini, B. Dozza, F. Rossi, M. Fantini, A. Spadari, N. Romagnoli, E. Landi, A. Tampieri, A. Piattelli, G. Iezzi, R. Scotti

**Affiliations:** ^1^Section of Prosthodontics, Department of Biomedical and Neuromotor Science, Alma Mater Studiorum University of Bologna, Via S. Vitale 59, 40125 Bologna, Italy; ^2^Osteoarticular Regeneration Laboratory, Rizzoli Orthopaedic Institute, Department of Biomedical and Neuromotor Sciences, Alma Mater Studiorum University of Bologna, Via Pupilli 1, 40136 Bologna, Italy; ^3^Department of Biomedical Science and Neuromotor (DIBINEM), Alma Mater Studiorum University of Bologna, Via S. Vitale 59, 40126 Bologna, Italy; ^4^Private Veterinary Clinic, 40037 Sasso Marconi, Italy; ^5^Virtual Reality and Simulation Laboratory, Second Engineering Faculty, Alma Mater Studiorum University of Bologna, Via Fontanelle 40, 47100 Forlì, Italy; ^6^Faculty of Veterinary Medicine, Alma Mater Studiorum University of Bologna, 40064 Ozzano dell'Emilia, Italy; ^7^Institute of Science and Technology for Ceramics, National Research Council, Via Granarolo 64, 48018 Faenza, Italy; ^8^Department of Oral Sciences, University of Chieti, Via de' Vestini 31, 66100 Chieti, Italy; ^9^Section of Prosthodontics, Department of Oral Science, Alma Mater Studiorum University of Bologna, Via S. Vitale 59, 40125 Bologna, Italy

## Abstract

*Purpose*. This study evaluated the efficacy of a regenerative approach using mesenchymal stem cells (MSCs) and CAD-CAM customized pure and porous hydroxyapatite (HA) scaffolds to replace the temporomandibular joint (TMJ) condyle. *Methods*. Pure HA scaffolds with a 70% total porosity volume were prototyped using CAD-CAM technology to replace the two temporomandibular condyles (left and right) of the same animal. MSCs were derived from the aspirated iliac crest bone marrow, and platelets were obtained from the venous blood of the sheep. Custom-made surgical guides were created by direct metal laser sintering and were used to export the virtual planning of the bone cut lines into the surgical environment. Sheep were sacrificed 4 months postoperatively. The HA scaffolds were explanted, histological specimens were prepared, and histomorphometric analysis was performed. *Results*. Analysis of the porosity reduction for apposition of newly formed bone showed a statistically significant difference in bone formation between condyles loaded with MSC and condyles without (*P* < 0.05). The bone ingrowth (BI) relative values of split-mouth comparison (right versus left side) showed a significant difference between condyles with and without MSCs (*P* < 0.05). Analysis of the test and control sides in the same animal using a split-mouth study design was performed; the condyle with MSCs showed greater bone formation. *Conclusion*. The split-mouth design confirmed an increment of bone regeneration into the HA scaffold of up to 797% upon application of MSCs.

## 1. Introduction

Strategies for repairing large bone defects are increasingly oriented towards bone graft substitutes and bone-tissue-engineering scaffolds. Currently, there is great interest in tissue engineering, a multidisciplinary approach to tissue regeneration that integrates engineering principles with life science, bypassing the drawbacks of autografts and improving the allograft, xenograft, and alloplast properties [1–3].

Different strategies are adopted in designing a scaffold able to regenerate bone with good mechanical and functional properties such as biocompatibility, osteoconduction, bioactivity, osteoinduction, and biodegradation [4].

One of the most important challenges in tissue engineering is to obtain an environment for cellular attachment, proliferation, and differentiation.

For this purpose, three-dimensional (3D) porous materials are used. The 3D porous structure provides space for new bone formation, the support necessary for cells to proliferate and maintain their differential function, mimics many roles of the extracellular matrix, and its architecture defines the ultimate shape of new bone [5, 6].

Experimental temporomandibular joint (TMJ) condylar prostheses have been described in primates [7, 8], pigs [9], and sheep [10].

In the last decade, bone substitutes have been used in combination with osteogenic cells for prefabrication of bioartificial bone grafts in several animal studies [11–19]. The use of multipotent mesenchymal stem cells (MSCs) has generated new therapeutic approaches for bone substitution [20]. The scaffold can be implanted into the patient to function as replacement tissue after *in vitro *MSC colonization (tissue engineering) or may be seeded with MSCs during surgery (regenerative medicine).

Sheep have bone remodeling patterns similar to those of humans [21], with at least equal chewing forces. The TMJ undergoes adaptive changes even when chewing causes only minor displacement of the condylar head, while articular degenerative changes may develop earlier in adults because of their poor aptitude for subchondral bone remodeling. Therefore, CAD-CAM processing of computed tomography (CT) data for a perfect scaffold reproduction of the existing condyle (shape-geometry) is used [22–24]. Because of their prolonged daily mastication, the articular tubercles and eminences in sheep are thin, similar to human TMJs, and thus, from a biomechanical point of view, sheep are preferred.

We evaluated a new approach using a CAD-CAM customized pure and porous hydroxyapatite (HA) scaffold to replace the TMJ condyle. First, we analyzed the interaction between the scaffold and the surrounding tissues (bone and soft tissue) to better understand the biological properties of the scaffold (osseointegration and material degradation). Second, we examined qualitatively scaffold behavior both inside and at the joint surface.

Our main evaluation focused on the formation of new bone within the scaffold through measurement of the HA porosity, reduction of total pore area in the condyle sections, and the area filled with new bone with or without MSCs. We further evaluated the scaffold and cell behavior at the interface with the articular cartilage as well as the scaffold resorption capacity.

## 2. Materials and Methods

The study was approved by the Italian Minister of Work, Health and Social Policies Ethical Committee on March 11th 2009 (decree n. 51/2009-B).

### 2.1. Scaffold Fabrication

The HA scaffold was constructed from a commercial powder (Finceramica, Faenza, Italy). The pore size and distribution were optimized by establishing a foaming process based on aqueous suspension of the HA powder (30% volume of solid loading). A ball-milling process lasting 20 h was used to obtain the desired foaming characteristics. The foamed suspension was poured into designated parallelepiped molds and dried in an air-circulating oven for 48 h at 40°C. The samples were then sintered at 1250°C for 3 h. The sintered samples were characterized using X-ray diffraction (Cu K*α* radiation; Rigaku Miniflex, Tokyo, Japan), scanning electronic microscopy (SEM Stereoscan 360; Leica, Cambridge, UK), and Hg porosimetry (Porosimeter 2000 and Macropores Unit 120; Carlo Erba, Milano, Italy) to determine the physicochemical and morphological features. The material was phase pure stoichiometric HA, with a globular multidimensional porosity amounting to 65%–70% volume. Large 150–1000-*μ*m pores were interconnected by 70–120-*μ*m pores; smaller size micropores (no less than 10 *μ*m) were also present. Further, the mechanical potential of the new porous scaffolds before implantation was assessed. The scaffold was preliminarily tested under compression (Instron model 1195, High Wycombe, Bucks, UK) to compare the mechanical performance with similar porous HA developed and tested *in vivo*. The 3D porous materials used in this study (HA-F70) showed nearly twice the compressive strength of samples previously tested *in vivo* (HA-S45) and were also characterized by a markedly lower total porosity.

### 2.2. CAD-CAM Processing: Customization of the HA Scaffold

Starting from a stack of CT slices, the 3D digital model of the sheep mandible was reconstructed by setting a suitable threshold value using Amira 3.1.1 (Mercury Computer Systems, Chelmsford, MA, USA). Both condyles were then virtually resected from the model to artificially create a bone defect in the mandibular ramus after planning the cut line with the surgeon.

The healthy and resected mandibular rami were 3D printed with an acrylonitrile butadiene styrene (ABS) plastic material using a Stratasys Dimension Soluble Support Technology (SST) 3D printer (Stratasys Inc., Eden Prairie, MN, USA).

Surgical guides and fixation plates were custom-designed by Rhino 4.0 (Robert McNeel & Associates, Seattle, WA, USA). The surgical guides were designed to allow the surgeon to reproduce the virtually planned surgical mandible excision during the procedure. Customized reference-coupling elements were also designed to facilitate the correct positioning on the bone, and two 2 mm diameter holes were drilled for bone anchoring using titanium screws (Gebrüder Martin GmbH & Co. Ltd., Tuttlingen, Germany). The fixation plates were designed by thickening the outer ideal contour surface of the mandible to firmly secure the HA scaffolds to the bone and ensure primary stability during mastication. To ensure the correct position of the scaffold with respect to the resected condyle, two plate reference holes were exactly placed according to the fixing holes of the relative surgical guides. An array of 2 mm diameter holes arranged along the surface of the plates was also provided to facilitate blood supply to the graft material and to properly fix the HA scaffold to the plate using titanium screws.

Then, the surgical guides and the fixation plates were directly manufactured using an EOSINT M270 machine (EOS Gmbh Electro Optical Systems, Munchen, Germany). Surgical guides were manufactured in EOS Cobalt-Chrome MP1 and fixation plates in EOS Titanium Ti64, an alloy with excellent mechanical properties and corrosion resistance combined with low specific weight and biocompatibility.

A three-axis CNC subtractive automated milling machine (Cortini HS 644 P; Fidia S.p.A., Padova, Italy) was used to prepare the externally designed scaffold volume to replace the resected condyle. The 3D automated machining process was initiated from a parallelepiped block of porous HA of the required size (60 × 30 × 20 mm). A machining tool with a 3 mm diameter spherical milling bur was chosen and the machining process was conducted to build the final scaffold with a cut depth of 0.5 mm for the roughing step and 0.2 mm for the finishing step. Before connecting the HA scaffolds to the fixation plate with titanium fixing screws, holes were drilled with a diamond bur at 24,000 rpm with irrigation and an axial movement to avoid microfractures in the material. The anatomic landmarks for screws were made in the scaffolds before surgery using the prototyped ABS models of the mandibular rami with the same bone defects used for restoration. The enveloping shape of the customized plates allowed scaffold fixation with a single titanium screw. Finally, the composite specimens, bone-fixing titanium plate, and HA scaffold were sterilized with gamma rays (25 KGy) for 72 hours.

### 2.3. MSCs and Platelet-Rich Plasma Preparation

Under general anesthesia, a 10 mL sample of bone marrow was aspirated into a 20 mL plastic syringe from the posterior iliac crest, approximately 4 weeks before implantation. Bone marrow was collected by inserting the needle into multiple sites. The volume of aspirated bone marrow was transferred immediately to vacutainer tubes containing 10.8 mg K_2_EDTA as an anticoagulant (Becton Dickinson, Franklin Lakes, NJ, USA). Nucleated cells were isolated on a density gradient and resuspended in alpha-modified essential media (alpha-MEM; Sigma Chemical Co, St. Louis, MO, USA) containing 10% fetal calf serum (FCS; Euroclone, Wetherby, UK), 100 units/mL penicillin (Euroclone, Wetherby, UK), 100 mg/mL streptomycin, and 1% GlutaMAX (Gibco Invitrogen, Paisley, Scotland). All nucleated cells were plated in a 150 cm^2^ culture flasks and incubated in a humidified atmosphere at 37°C with 5% CO_2_. Nonadherent cells were discarded after 2 days and adherent cells were cultured for further expansion. When cultured flasks became near confluent, cells were detached by mild trypsinization (TripLe Select, Gibco, Invitrogen, Paisley, Scotland) for 5 min at 37°C and reseeded onto new plates at one-third density for continued passage. Media were changed every 3 to 4 days. Cell number and viability were assessed at each passage and in each experiment using NucleoCounter (Chemometec, Allerod, Denmark) and were consistently over 98%.

Platelets were obtained from the venous blood of the sheep. Blood was drawn from the jugular vein into a bag containing CPD (327 mg citric acid monohydrate, 2.63 g sodium citrate dehydrate, 2.55 g glucose monohydrate, and 251 mg sodium dihydrogen phosphate dihydrate per 100 mL of CPD) as an anticoagulant (1 mL CPD/7 mL blood). Blood was centrifuged twice: the first time at 1200 rpm for 40 min at 20°C to remove red blood cells and the second at 4000 rpm for 10 min at 20°C to obtain platelet-rich plasma (PRP), resulting in a platelet number of 1 × 10^6^ /mL. When 330 *μ*L of calcium gluconate (100 mg/mL) was added to 10 mL of plasma, autologous thrombin was released. Mixing 4 mL of thrombin with 16 mL of PRP caused platelets to release their granular content, and the platelet gel was thus obtained. The final platelet concentration was evaluated to be approximately 8 to 10 times the initial sample count.

On the day of the surgical procedure, cells were detached from the culture dishes and mixed with the carrier composed of 1 : 1 platelet gel and sterile rat tail-derived collagen (Roche GmbH, Mannheim, Germany), to achieve a concentration of 4 × 10^6^ cells per 1 mL of scaffold. The PRP-based carrier containing the cells had a gel consistency. The gel was injected around the implant at the end of the procedure immediately before soft tissue closure.

### 2.4. Animal Housing and Anesthetic Procedures

Each sheep mandible was examined by CT using a 16-slice CT scanner (Brightspeed GE Medical System, Milwaukee, WI, USA) with the animal under general anesthesia: sedation was induced with ketamine (5 mg/kg intramuscularly (IM)) and midazolam (0.4 mg/kg IM); induction was achieved with propofol (3 mg/kg intravenously (IV)) and ketamine (1 mg/kg IV). CT scanning was performed in axial sequence using 140 kV/315 mAs with a slice thickness of 0.625 mm, an interval of 0.3 mm, a reconstruction algorithm for bone, and an image resolution of 512 × 512 pixels. Multiplanar and volume rendering reconstructions were obtained. A 2-year-old, nonpregnant, 48 kg female sheep was used in the present study, which was authorized by the ethics committee of the University and of the State. Before and after the surgery, the sheep was housed in a 3 × 3 m stall with straw and was fed hay. A 1-day fast with no water limitation was prescribed before the surgery. Surgery was performed under general anesthesia. Ketamine (5 mg/kg IM) and midazolam (0.4 mg/kg IM) were administered for sedation. After 20 min, the cephalic vein was catheterized, fentanyl (4 *μ*g/kg IV) was administered to provide analgesia, and a patch of fentanyl (100 *μ*g/h) was applied before the surgical procedure to provide analgesia throughout the postsurgical period. Induction was achieved with propofol (3 mg/kg IV) and ketamine (1 mg/kg IV). After intubation, anesthesia was maintained with isoflurane (1.5% in a mixture of oxygen FO2 = 0.6 and a continuous rate infusion (CRI) of ketamine (1-2 mg/kg/h) and fentanyl (15 *μ*g/kg/h). After recovery, flunixin meglumine (1 mg/kg IM) (Meflosyl Intervet Schering-Plough) was administered to the animal every 12 h. As a perioperative prophylaxis measure, procaine benzylpenicillin (8000 IU/kg) and streptomycin (10 mg/kg IM UI SID) (Combiotic; Fatro, Ozzano, Bologna, Italy) were injected on the day of the intervention and the following day. The sheep showed no apparent pain during the postoperative period and started chewing movements immediately after recovery; rumination was observed after 8 h.

### 2.5. Surgical Procedure and Followup

A longitudinal 5 cm incision directly on the caudal margin of the ramus mandibulae, using the TMJ as the dorsal starting landmark, was performed. The posterior rim of the mandible was identified and the temporal muscle was detached from the bone. Muscle, as well as the buccal and zygomatic branches of the facial nerve, was pulled down leaving sufficient access to the entire superior mandible ramus. Then, the first surgical guide was applied using two titanium fixing screws. The condylar neck was divided with an oscillating saw following the line of the surgical guide below the insertion of the lateral pterygoid muscle. The TMJ was opened by cutting the capsule and leaving the meniscus on site. After the resection, the proximal fragment was dislocated and ablated. The surgical guide was freed and the bone prepared to host the scaffold.

The HA scaffold was prepared previously with the bone plate being connected. The same screw holes drilled to fix the surgical guide were used to position and secure the plates along with the HA scaffold. Fixation was achieved with bicortical screws (9 and 11 mm) and the insertion of the lateral pterygoid muscle was preserved. The MSCs were randomly seeded on the left or right side.

The animal was cared for at the Faculty of Veterinary Medicine and recovered in the stables for 16 weeks. Food and water were not rationed. The animal was sacrificed after 4 months with ketamine (5 mg/kg IM), midazolam (0.4 mg/kg IM), and an IV injection of embutramide (70 mg/kg) plus mebezony (15 mg/kg) (Tanax). CT examination of the skull after the sacrifice and before the harvest of the specimen was performed to compare the anatomic results in order to detect the positional variation after functioning with respect to the planned position at the condyles. The specimens were then explanted for the anatomical and histological examination. The mandibular HA condyles and the surrounding bone tissue of the ramus with adjacent connective tissue (in the articular area) were harvested as a single block. Specimen collection was performed immediately after the CT examination; the sample was submerged in 10% buffered formalin and delivered to the laboratory for histological analysis.

### 2.6. Histological Preparation

The specimens and surrounding tissues were processed to obtain macroscopic sections. To visualize the sectioned condyle surface, two main sections of the specimen were prepared: one parallel to the long axis of the ramus with a direction from the posterior (caudal) to the anterior part of the scaffold and the other parallel to the frontal plane from the lateral to the medial part of the scaffold articular surface. An irrigated continuous circular saw was used to separate the specimens, which were processed using an automated system (Precise 1 Automated System, Assing, Rome, Italy), dehydrated in a graded series of ethanol rinses, and embedded in a glycol methacrylate resin (Technovit 7200 VLC, Kulzer, Wehrheim, Germany). After polymerization, the specimens were sectioned parallel to the main section with a high-precision diamond disk at approximately 150 *μ*m and ground down to approximately 30 *μ*m with a specially designed grinding machine. Three slides were obtained from the long axis parallel to the ramus (scaffold core), and two slides were obtained from the long axis parallel to the frontal plane (condyle surface). The slides were stained with acid fuchsin and toluidine blue and examined under transmitted light with a microscope (Leitz, Wetzlar, Germany).

### 2.7. Histological Assessment

Histomorphometric analysis was performed by one experienced investigator blinded to the specific experimental condition, using a Leitz Orthoplan Light Microscope (LM, Leica Microsystem Inc., Bannockburn, IL, USA) equipped with a computerized image analyzer system (Qwin, Leica Microsystem Imaging Solution Ltd, Cambridge, UK). Three sections from each sample were chosen randomly and observed under LM for histomorphometric analysis. Five photographs were taken from each section. A region of 1218 × 898 *μ*m (1.09 × 10^6^ *μ*m^2^) including porous HA was selected as the standard region of interest (ROI). For each photograph at a final magnification of 6.3x, the Leica Qwin software provided the porosity percentage (percentage of area of voids over total area of each sample) and bone ingrowth (BI; the amount of new bone growth inside the HA pores (New Bone/ROI)) values.

The total pore area inside the HA sample was traced and the area filled by newly formed bone was detected using the Leica Qwin software. From these measurements, the percentage of HA porosity in the examined areas (area of pores/area of examination), the percentage of pore area filled with new bone (total ROI, residual voids areas/pores area), and the consequent reduction of the available porosity (porosity area, residual voids area) were obtained. The samples were analyzed as individual units (absolute value) for the effect of the presence or absence of MSCs on growth and bone maturation. Another observation included the analysis of the bone growth in the same animal for the presence or absence of cells in a condyle (test group versuscontrol group) to evaluate the difference caused by the MSCs in the same subject.

### 2.8. Statistical Analysis

Statistical evaluation of data was performed using the GraphPad InStat software, version 3.05 for Windows. Statistical analysis was conducted using Student's *t*-test, and *t* values were calculated with 10 degrees of freedom; the significance threshold was *P* = 0.05 and the confidence interval for a difference was 95%.

## 3. Results

### 3.1. Porosity (Figure 7)

**Figure 1:**
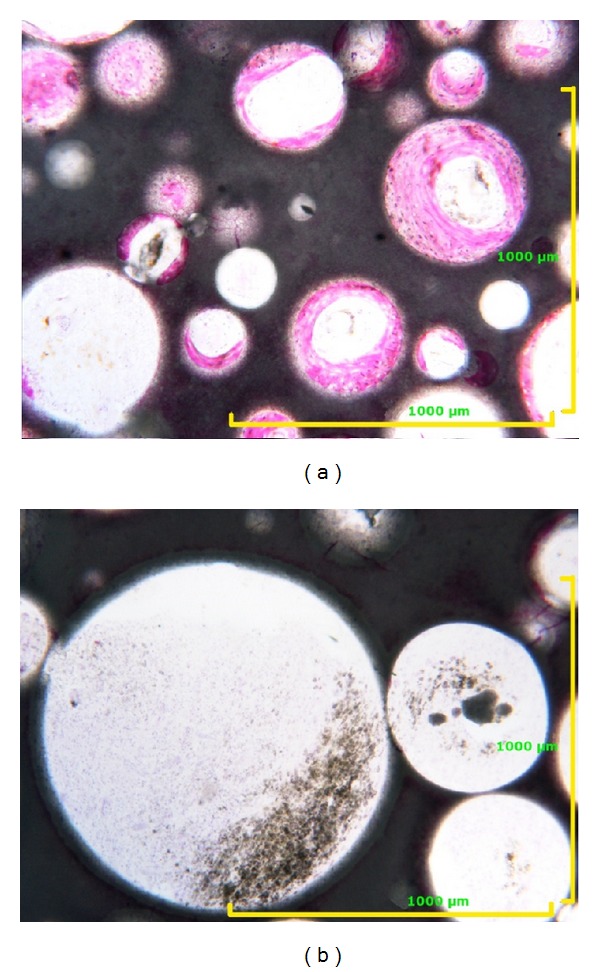
Examples of different porosity at the same analysis area: (a) 41% porosity. Toluidine blue acid fuchsin 40x; (b) 78% porosity. Toluidine blue acid fuchsin 100x.

The porosity values of the scaffold used were not homogeneous (Figure 1) and were calculated as the percentage of void area over the total area of examination for 120 areas of analysis (five areas in three different standardized positions for each condyle using four sheep). The values ranged from a minimum (14%) to a maximum (91%) level of porosity. According to a Gaussian distribution of the porosity percentage, the extreme values represent only the curve codes, while the mean frequency of porosity was 45.43%. This value was different from the total porosity of the material (70%) because it represents only the mean porosity value in the specimens used for histomorphometry. 

### 3.2. BI Absolute Values

Absolute values of MSC-seeded versus nonseeded condyles were based on specimens collected in six animals implanted bilaterally. Two condyles, one with and one without MSCs, were excluded from the analysis due to complications during the healing period. One condyle was excluded due to infection and the other because of a screw loosened during followup, causing displacement of the condyle. Individual condyles were assessed as a single unit in the comparison of MSC presence or absence referred to as “S” and “N”, respectively, in Table 1 and Figure 5, and the percentage of the HA porosity, the porosity reduction due to the bone filling, and the relative area of newly formed bone within the pores were calculated (Table 1). Analysis of the reduction in porosity due to the apposition of newly formed bone showed a statistically significant difference (*P* < 0.05) between condyles loaded with MSCs and condyles not loaded.

**Figure 2:**
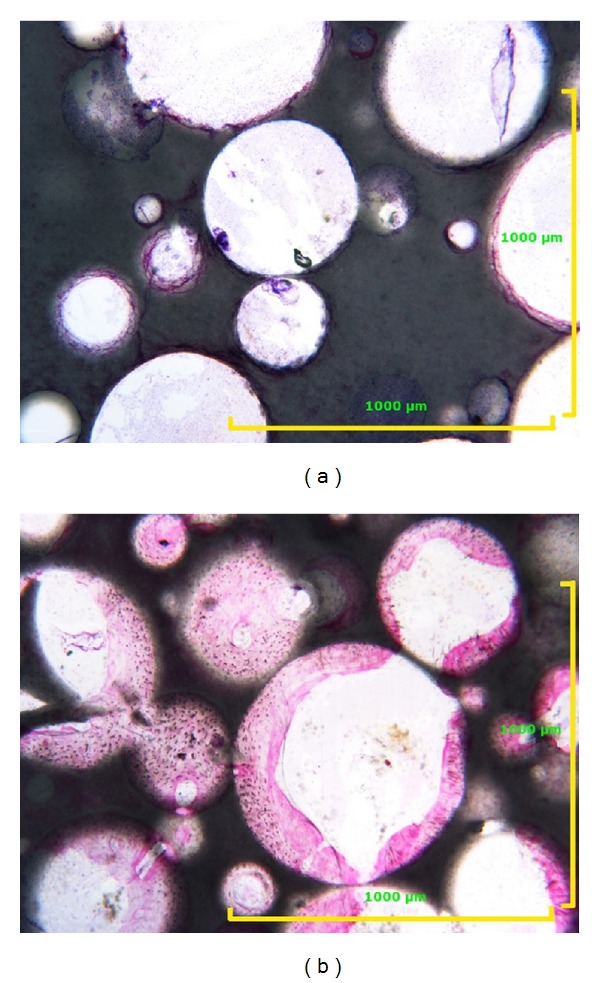
Example of two scaffold specimens with similar porosity: (a) without MSCs and (b) with MSCs. Toluidine blue acid fuchsin 400x.

The BI mean area in condyles without MSCs was 112.692 *μ*m^2^ (±95.787 *μ*m^2^), while that for condyles with MSCs was 315.501 *μ*m^2^ (±113.547 *μ*m^2^); this difference was statistically significant (*P* = 0.0389) (Figure 5). In the condyles without MSCs, the sample qualitative analysis demonstrated a bone maturation delay after a 4-month healing period, which was in contrast with the good organization and maturation of osteoblastic and osteoclastic cells in condyles with MSCs. The presence of MSCs accelerated and facilitated entry of the seeding mechanism into the pores and of new bone formation within the scaffold, augmenting the percentage of bone filling in the pores by a mean value of 180% (Figure 2).

**Figure 3:**
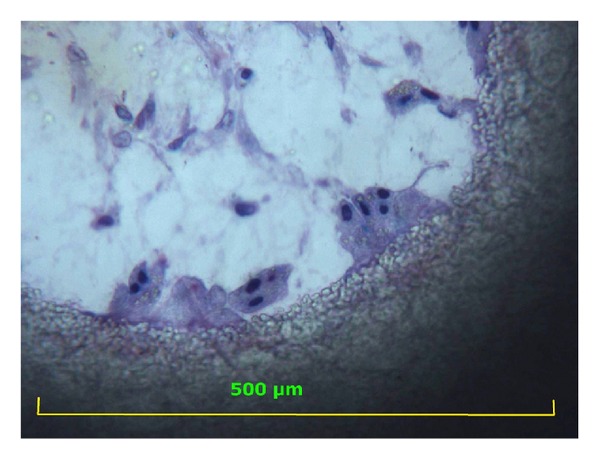
Higher magnification (400x) of multinucleated cells (osteoclasts) with polarized nuclei in the opposite site of the lysosomal activity while degrading the pore surface (note the HA fragment in the cytoplasm). Toluidine blue acid fuchsin 400x.

Scaffold resorption or remodeling was very limited because of the partial initial surface degradation due to lysosomal activity of polynucleated (osteoclasts) cells (Figure 3). The osteoblastic rim was evidently distant from the scaffold surface that was completely covered by mature bone with limited lysosomal activities [25]. However, the pores appeared deeply invaded from vessels during earlier bone formation, indicating the scaffold ability to be perfused by the organic fluids and vascularized, thus emphasizing the importance of a multiscale (micro to macro) interconnected porosity. In one of the two condyles excluded by the split-mouth study, the fractured HA had fallen into the medial muscle fascia and showed no involvement of the surrounding soft tissue. This was probably due to the lack of functional movement or physiologic use of the scaffold, thus no stimulus improved the penetration and differentiation of progenitor cells into the pores.

### 3.3. BI Relative Values

The BI relative values of split mouth were compared (scaffold with MSCs versus scaffold without MSCs). As explained previously, two animals were excluded from the split-mouth analysis. Differences between the condyles with or without MSCs were also identified by analyzing the same animals (test and control sides), using the split-mouth design of the study. The condyle with MSCs showed higher bone formation in the same animal.

Sheep 1 showed an area of new BI equal to 257937 *μ*m^2^ with MSCs and 112692 *μ*m^2^ without MSCs (129% difference). Sheep 2 showed a BI of 458210 *μ*m^2^ with MSCs and 51064 *μ*m^2^ without MSCs (797% difference). Sheep 3 showed a BI of 489576 *μ*m^2^ with MSCs and 65774 *μ*m^2^ without MSCs (644% difference). Sheep 4 showed a BI of 315501 *μ*m^2^ with MSCs and 260072 *μ*m^2^ without MSCs (21% difference, Figures 6(a), 6(b), 6(c), and 6(d)).

**Figure 4:**
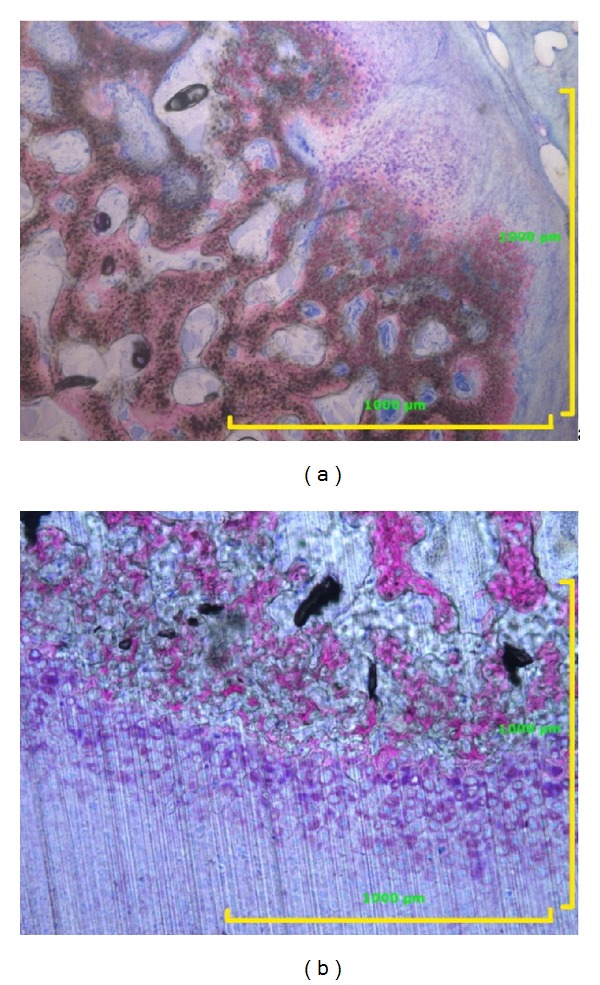
(a) Presence of cartilage at the condyle articular surface in sheep 3. Toluidine blue acid fuchsin 25x. (b) The medial portion of the MSC-seeded scaffold was completely covered by newly regenerated bone and cartilage at the top of the condyle. Toluidine blue acid fuchsin 100x.

**Figure 5:**
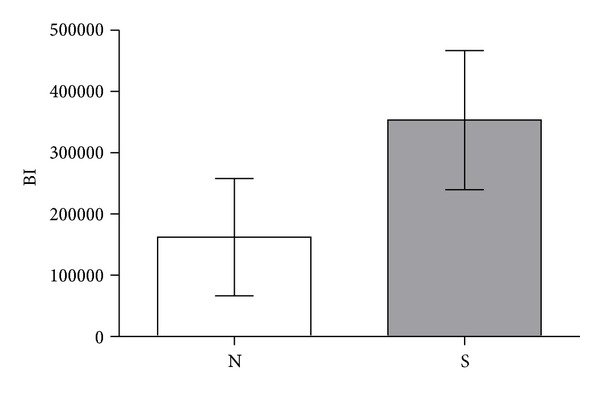
Mean MSC influence values.

**Figure 6:**
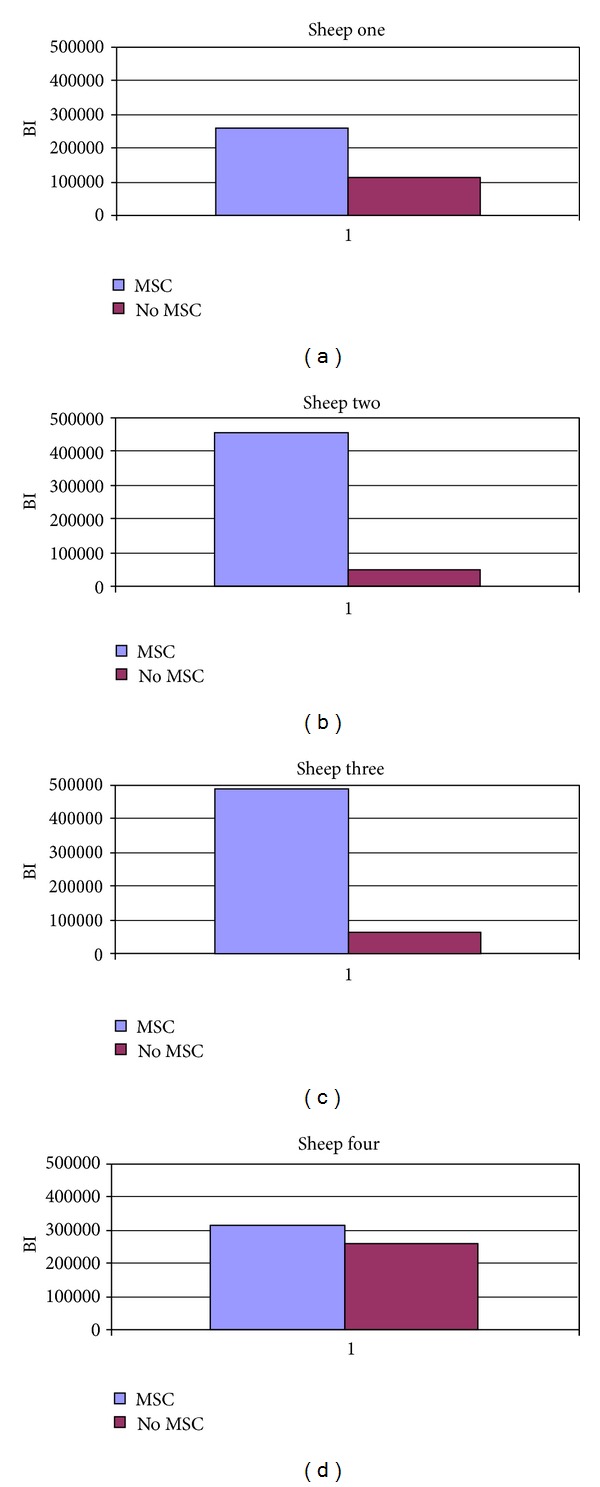
Split-mouth design: scaffold with MSCs versus scaffold without MSCs in each animal.

The macroscopic explant qualitative analysis in two samples showed a medial portion of completely new BI attached to the medial portion of the scaffold; that is, the entire medial wall of the ramus was regenerated. At the upper level of this medial regenerated portion of bone, a part of a new condyle was visible, reproducing exactly the bone-cartilage structure of the condyle (Figures 4(a) and 4(b)).

## 4. Discussion

Critical bone defects created after the resection of neoplasm represent an obstacle in regenerative medicine. Several materials are available: between the nonresorbable ones, the Poly-Ether-Ether-Ketone (PEEK) is a well-known material for bone substitution of large or small defects [26–29]. It showed no complications in comparison to the alloplastic implants (i.e., expanded polytetrafluoroethylene, porous polyethylene, methyl methacrylate, and silicone rubber) that presented postoperative complications, as swelling, infection, foreign body reaction, and displacement or extrusion [30–33]. However, no data are available about long-term maintenance and about biomechanical properties of PEEK when used in large defects with significant functional loading. 

Research in tissue engineering is focused on finding new approaches for bone regeneration that optimize results by seeding MSCs on a porous ceramic scaffold. The success of bone growth due to the stability and porosity of the material has been reported widely [20–24, 34]. Several orthopedic studies in large animals emphasize the difference between the healing of sites treated with scaffolds loaded with MSCs compared to scaffolds without MSCs [35–42].

Kon et al. [37] evaluated use of MSCs to repair bone defects in a sheep model. They reconstructed a critical defect in the tibial bone using two methods: a porous scaffold of 100% HA seeded with MSCs and the scaffold alone without MSCs, with the empty defect as the control. Even though bone formation was observed histologically in both groups, in the seeded scaffolds bone formation occurred both within the internal pore space and around the scaffold, while, in the HA without MSCs, bone formation was limited to the surface and was not observed in the majority of the inner pores.

Petite et al. [24] used a similar sheep model with *in vitro* expanded MSCs in a resorbable coral-based scaffold and reported that host-scaffold union did not occur when the defects were left empty or filled with scaffold alone. In a further study [38], the same group suggested that the modality of cell seeding over the scaffold influences the bone-forming ability of the implant. Yuan et al. [40] reported a study in which a 30 mm long mandibular segmental defect created in a canine model was repaired using osteogenically induced MSCs seeded on porous beta HA. Five animals were implanted. In the experimental group (MSC group) new bone formation was observed 4 weeks postoperatively, and a bony union was achieved after eight months. Conversely, minimal bone formation with very poor connection occurred in the group treated with the scaffold alone. Schliephake et al. [42] used autogenous cultivated osteoprogenitor cells in porous calcium-phosphate scaffolds; these increased bone formation in a 35 mm segmental defect in the mandible of eight sheep. The authors reported significantly greater bone formation than in the group with cultivated bone cells. Bone formation was present in 34.4% of the evaluated cross-sectional units in the seeded scaffold but in only 10.4% of the control group. Moreover, a significant difference was observed in the spatial distribution of the bone formation across the scaffold; osteoprogenitor cells appeared to have increased bone formation, particularly in the center of the defect when compared to the control group.

Although these studies differ with regard to the animal model used, the type of defect, the features of bone treated, the chemical composition and the resorption ability of the scaffold used, in all samples of MSC-seeded scaffold, a significant advantage in bone formation, and healing of the defect were found. 

In our study, a split-mouth model was selected because of its statistical value even in a small population. From a quantitative point of view, two types of analyses were performed. The first analyzed the data as absolute values, without considering whether the results came from the same animal, accounting only for whether MSCs were present or not. The second analyzed the data as relative values, using the split-mouth experimental study design.

Moreover, our model used an articular defect instead of intercalary defects, as in the previously mentioned studies, to describe the regenerative process in a more complex defect as in the osteocondral surface of the TMJ and to account for all potential orthopedic scenarios using scaffolds in articular areas.

Our data contain a degree of heterogeneity with different degrees of bone formation among animals, most likely due to the local individual host response. Indeed, the seeded MSCs can exert a paracrine effect and can recall several healing enhancers (osteoprogenitor cells) that are individually variable [34–36]. According to reports concerning the effect of pore size, smaller pores played a more important role in initial cellular anchorage and attachment than larger pores [36]. In our study, the marked porosity variation among the specimens did not allow for detection of a correlation between cell seeding and pore size. However, albeit with different degrees of probability according to pore size, scaffolds seeded with MSCs always showed a greater BI.

When the scaffold achieved primary stability during the immediate followup, a continuous bone-scaffold union was observed with no interposition of connective tissue. The HA structure and the superficial composition allowed perfect osseointegration at the bone-scaffold interface. The importance of a stable mechanical condition was confirmed by the condyle that fractured and fell into the medial muscle fascia, which showed no integration with the surrounding tissues.

The second analysis compared the two condyles (with and without MSCs) in the same animal, according to the split-mouth design of the study using four sheep.

### 4.1. Analysis of Absolute Bone-Filling Values

In the quantitative analysis of the bone filling of the HA pores, the mean new bone value at the inner pores of the MSC-seeded scaffolds was significantly different than that of scaffolds without MSCs. This difference was important because the mean area showing BI at the inner HA pores was 315.501 *μ*m^2^ for the MSC group, while that in the control group was 112.692 *μ*m^2^. This result confirmed the literature data regarding the potential of MSCs in bone regenerative medicine. Since the standard deviations (SD) varied markedly (MSC group SD = ±113.547 *μ*m^2^ and control group SD = ±95.787 *μ*m^2^), a more detailed analysis of the split-mouth data comparison was conducted to evaluate the increment of bone filling upon use of MSCs in the same animal.

### 4.2. Bone-Filling Analysis in the Split-Mouth Model

In the four animals included in this section of the study, the increment percentage values due to MSCs ranged from 21% to 797%. This wide range was due to the individual host response and to surgical and follow-up factors. The sheep with the lowest increment value (sheep 4) was affected by a severe left parotid gland inflammatory reaction during the second month of healing and was treated with anti-inflammatory drugs and antibiotics for 1 week. Inflammatory cells were also found in the left scaffold condyle. The MSC-seeded scaffold side was affected. Sheep 1 had a partial fracture at the basis of the right scaffold (MSC-seeded), probably causing a micromotion at the interface that interfered with the bone healing process around and within the scaffold. 

A further qualitative observation indicated that the articular capsules were restored and perfectly functioning in all specimens except the two unsuccessful samples. Macroscopic analysis of the scaffold explants showed an intact but unusual articular chamber, with the articular disc firmly adhered to the condyle articular surface such that the chamber was not divided into two areas (upper and lower, divided by the disc). The glenoid fossa was intact, and no signs of inflammation were present after 4 months of healing. Synovial fluid was also present, and the condyle ligaments appeared to be correctly connected to the posterior, medial, and anterior areas of the capsule.

An interesting observation was the presence of new cartilage on the articular surface of the regenerated bone. Medial to the scaffold (MSC side) of two right condyles, a completely new regenerated bone wall was detected, and on its articular portion, new cartilage was discovered. The new cartilage on the medial portion of the new condyle ended where the scaffold surface (with the adhered disc on top) started (Figures 4(a) and 4(b)). Further studies will be necessary to determine why and under what conditions the new bony wall developed and new cartilage grew on the regenerated bone. 

Further studies are necessary to evaluate new mandibular bone shapes for the reconstruction of the mandible body and to allow innovative developments in the chemistry of the material used for scaffolding.

## 5. Conclusions

The result confirmed the literature data regarding the potential of MSCs in bone regenerative medicine. The split-mouth design showed an increment of bone regeneration into the HA scaffold of up to 797% upon application of MSCs. Macroscopic analysis of the scaffold explants showed an intact but unusual articular chamber and the histological analysis documented new cartilage on the articular surface of the regenerated bone.

## Figures and Tables

**Figure 7:**
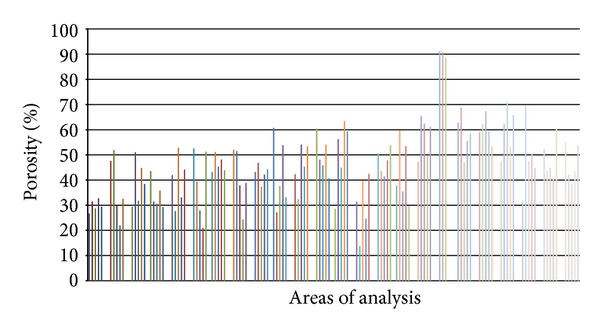
Porosity values of each examination area.

**Table 1:** Analysis of the influence of MSCs on all specimens (condyles) as a single unit.

Condyle	MSC	HA porosity	Porosity reduction	New bone area
1 N		35.20%	14.60%	5.30%
2 N		43.30%	31.90%	14.10%
3 N		45.90%	13.20%	5.90%
4 N		58.50%	6%	3.40%
5 N		47.90%	28.50%	13.50%
1 S	X	37.50%	31.10%	12.80%
2 S	X	42.80%	28.20%	13.40%
3 S	X	54.20%	44.30%	23.90%
4 S	X	55.70%	45.20%	25.50%
5 S	X	59.50%	28.20%	16.40%
